# Retraction: Overexpressed PKCδ Downregulates the Expression of PKCα in B16F10 Melanoma: Induction of Apoptosis by PKCδ via Ceramide Generation

**DOI:** 10.1371/journal.pone.0333839

**Published:** 2025-10-06

**Authors:** The PLOS One Editors

After this article [1] was published, concerns were raised regarding results presented in Figs 1-5.

Specifically:

- The following lanes appear similar:
  - Fig 2C PLD1 lane 1 and Fig 4B pAKT lane 3
  - Fig 2C PLD1 lane 3 and Fig 4B pAKT lane 4
  - Fig 2C PLD1 lane 4, Fig 2C PLD1 lane 6, and Fig 4B pAKT lane 2
  - Fig 2C PLD1 lane 5 and Fig 4B pAKT lane 1
  - Fig 3A AKT lane 1 and Fig 3A AKT lane 4
- The following panels appear to fully or partially overlap:
  - Fig 1D GAPDH, Fig 4C GAPDH, and Fig 5A GAPDH
  - Fig 3A GAPDH and Fig 4B GAPDH
  - Fig 5A Caspase 8 and Fig 5A Caspase 3
- The following results appear similar:
  - Fig 3B PKC δ siRNA panel and Fig 3D PLD1 siRNA + PKC α OV panel
  - Fig 3B EV panel and Fig 4A EV panel

First author KH stated that the underlying data are no longer available.

In light of the above concerns that question the reliability of the reported results and conclusions, the *PLOS One* Editors retract this article.

KH and AB agreed with the retraction. SB, SaikatM, and SubrataM did not respond directly or could not be reached.
